# Surgical Essays; the Result of Clinical Observations Made at Guy's Hospital

**Published:** 1834-01-01

**Authors:** 


### MEDICAL QUARTERLY REVIEW.

### REVIEWS.

*Surgical Essays; the Result of Clinical Observations made at Guy's Hospital.* By B. B. Cooper, F.R.S., Surgeon of Guy's Hospital, &c.—London, 1833. 8vo. pp. 281, and 4 plates.

The old division of things into good, bad, and middling, though somewhat of the tritest, seems to us to be especially applicable to the classification of medical books. First in order come the works of genius, which the generous superstition of the ancients was ever ready to attribute to the immediate inspiration of the gods: such as those of Hippocrates, which could no more have been achieved in this fidgetty age, when everyone is harassed with business, and overwhelmed with practice, than a Nash or a Wilkins could have planned the pyramids of Egypt. Next to these, but with a long interval between, come those sober but useful works, the offspring of some talent and much experience, which teach the application of the rules that genius has discovered. Lastly, we must mention the small fry of medical literature, those squeezings out of nothing, which produce little but advertisements, and please no one but the trunkmaker. Now, as it would be unjust to place Mr. Cooper's work in the last class, it would be ridiculous to rank it as one of the first: it is an honest, homely, practical book; and, though we could have wished that some literary friend had put it into English, in these degenerate times we must be thankful for what we can get, and confess that these essays will be useful, especially to beginners.

The book commences with a few remarks on "the Physiology of the Growth and Reparation of Bone." Our author says,

"The hardness which forms the great characteristic of bone too frequently leads the surgeon to consider this physical property as separating the osseous system, and the phenomena attending the diseases incident to it, entirely from the same vital influence experienced in the changes attending the diseased actions of the softer parts. But, upon investigation, it will be found that the same laws preside in both instances; modified, however, in the former, by the presence of a proportion of earthy matter, which differs in quantity not only in different bones, but in separate parts of the same bone.

"By many eminent surgeons this earthy matter has been considered and described as being inorganized; but it is hardly to be conceived that dead matter can exist in connexion with living, without producing the ill effects experienced by the presence of all extraneous substances." (P. 3.)

What surgeon ever thought that the bones of living persons were not alive? And what eminent surgeons ever described the phosphate of lime in the bones as being inorganized matter? We are tempted to cry, Name! name! as they do when incredible statements are broached in a certain great assembly.

Our author's account of the progress of reunion in fractured bones is deficient in precision and exactness. He says, "where the periosteum is extensively lacerated, the production of bone still goes on, partly from the denuded surface, and partly from the surrounding soft structures, so that the common cellular tissue is convertible into periosteum, or a substance capable of performing the same function, when in contact with the granulations arising from bone, which it cannot be until the periosteum has been destroyed. Thus, periosteum may be considered as not only useful in distributing its blood-vessels for the formation of bone, but also as constituting the limit between the osseous and other tissues." (P. 9.)—[*Limit*, quotha! the author must mean *link*.]

Mr. B. B. Cooper would have done well to mention that, except in the rare case of partial fracture, or fracture within the periosteum, this membrane does not contribute to the reparation of bone; and, even in that particular instance, its agency is evidently of little moment. Some years since, our honoured predecessor, the "London Medical and Physical Journal," contained an account of some experiments by Mr. Mayo, in which the common source of reparation, whether in bone, cartilage, nerve, or tendon, was shewn to be in the thickening of the surrounding tissues to form a callus. The most unexpected part of the process is, that the callus, which first mechanically glues together the separated ends of the fractured part, originates the final and textural reparation. We might also remark, that at p. 11, where our author treats of fractures of the flat bones, he has omitted to mention the curious peculiarity in the reparation of fractures of the cranium: the student ought to have been told, that here no callus is formed, but the injury is repaired by a direct though very tardy extension of the osseous substance itself.

In speaking of fractures of the pelvis, Mr. Cooper says,

"The surgeon should next place the patient in a perfect horizontal posture, and examine if the anterior and superior spinous processes are on a level, and if the lower extremities are of the same length; when a want of symmetry in either of these instances would necessarily indicate displacement of the bones of the pelvis. I should here recommend, before the surgeon attempts to discover which of the bones of the pelvis has sustained the injury, that he should first pass a catheter into the bladder, to discover whether or not the urethra has been injured; for, in the force necessary to ascertain the seat of injury to the bones, an additional laceration of the urinary apparatus might be inflicted, and which may be avoided by having ascertained, in the passage of the catheter, either the safety or injured state of these parts." (P. 15.)

But surely a surgeon cannot be justified in using such force as might produce an additional laceration of the urinary apparatus; particularly since, even if he succeeds in ascertaining the exact direction of the fracture, he can do nothing more than if he vaguely suspected a fracture to exist: in such a case, rest and a bandage are all that surgery can supply.

"The following case," says our author, "is somewhat singular, from there being no fracture of the bones of the spine, but a laceration of the intervertebral substance, admitting of such a displacement of the vertebræ as to press upon the spinal marrow; producing therefore precisely the same symptoms, and leading completely to the same practice, as if fracture had occurred; thus rendering it quite unnecessary to describe this case under a separate head.

"Case. Joseph York, a stout man, aged forty-five, was admitted on Monday, April 8th, 1833, at half-past nine p. m., with injury to the spine, occasioned by a barrowful of grains falling a height of fourteen feet upon his neck and head, producing three severe lacerations of the scalp, and an immediate loss of all sensation and motion of his lower extremities. He was directly brought to Guy's Hospital, which was in the vicinity, the accident having happened at Barclay's brewhouse; and on admission the symptoms were as follow:

"Paralysis of all the intercostal as well as the abdominal muscles, so that respiration was carried on completely by the diaphragm. He had partial priapism, tympanitic abdomen; complained of general sensation of coldness, although the surface was naturally warm. His pulse were only fifty-six, and labouring. There were three lacerated wounds on the scalp, one of which was sufficiently deep to divide the bone, but as he was perfectly sensible, there was sufficient evidence that the symptoms did not arise from injury to the brain; he was therefore placed in the prone position to examine the spinal column, when it was found that, on pressure over the sixth and seventh cervical vertebræ, there was some motion of these bones, that pain was felt extending upwards, and was increased by pressure.

"The head was ordered to be shorn, and the wounds dressed; as there was considerable injury of the soft parts about the injured spine, a cupping-glass was applied on either side, and about four ounces of blood withdrawn.

"His water was drawn off with a catheter, and was found to be very acid, as proved by reddening litmus.—The Julep. Ammon. was desired to be given at intervals.

"9th. Eight A.M. Has had no sleep during the night; complains of some pain and difficulty of motion in his arms, although he describes himself altogether as feeling better; has neither voided urine nor fæces since his admittance; his respiration is quick and difficult; pulse seventy-two, increased in power, and less labouring. His urine was drawn off, and was still found acid. The priapism still remains.—Ordered common enema stat.

"Two P.M. Continues much in the same state. The enema has not acted; ordered to be repeated. Still complains of sensation of coldness.

"Nine P.M. It has been necessary to administer a third enema, which at present has not had the desired effect. He is now much worse in every respect; the breathing quick, short, and irregular. The abdomen was more tympanitic. His urine was again drawn off; is still acid.

"10th. On this morning's report, it was found his bowels had been freely opened; of which he was perfectly unconscious. His breathing slower, but more difficult; the pulse full and soft. The surface of the body was now cold, and clammy to the touch; his countenance anxious, although he retained completely all his powers of mind, and seemed fully conscious of his approaching dissolution.

"At twelve M. an evident change had taken place since last report, and from this time he gradually sunk, and died at three P.M.

"About twenty hours after death the body was examined; and, on exposing the cervical vertebræ, a complete laceration of the intervertebral substance between the fifth and sixth was discovered, and without any fracture. The capsular ligament of the articulation between the two vertebræ on the left side was torn through.

"On removing several of the vertebræ with the spinal marrow, and then examining the spinal chord, a slight blush of inflammation was found on the inner surface of the dura mater, but no laceration or lesion of the medulla spinalis." (P. 46.)

The symptoms were not "precisely the same." When the spinal chord is crushed, the urine is alkaline: in this case there was "no laceration or lesion of the medulla spinalis," and the urine was acid. Mr. Cooper says that the patient's urine "was found to be very acid, as proved by reddening litmus." Erase the *very:* the weakest acid reddens litmus.

Immediately after discussing fracture of the sternum, our author gives an interesting case which occurred in the practice of his great namesake and relation:

"It sometimes happens that inflammation extends into the anterior mediastinum, affecting the absorbent glands, or perhaps the remains of the thymus gland itself, which may go into a suppurative state, producing dyspnœa, from pressure upon the lungs; disease of the sternum, from its tendency to be evacuated externally; and presenting a pulsating tumour, from its contiguity with the heart.

"Such a case occurred in the practice of Sir Astley Cooper, in the person of a medical student, who believed himself, and had been led to believe by other medical men, that he was the subject of aneurism; under which conviction he had made up his mind to make a voyage to Bourdeaux, there, as he believed, to die. Before he left London, however, he thought he would consult Sir Astley Cooper, who told him at once the nature of his complaint, evacuated the matter, which had made its way through the sternum, and removed simultaneously both the appearance of aneurism and the fears of the patient." (P. 59.)

Under the head of "Fractures of the Short Bones," our author mentions a case of extensive fracture of the bones of the face, which appears to us worth extracting.

"Martha Ambrose, aged forty-eight, was admitted into Guy's Hospital, March 29th, 1829, for an injury which she had just received from being thrown down by a horse which had run away with a gig. Upon her admission, a more deplorable object could scarcely be conceived: all the soft parts on the right side of the face were detached from the bones, which were most extensively fractured; the lower jaw was fractured in two places; the superior maxillary and palate bones were broken through their palatine processes, so that the roof of the mouth fell upon the tongue, and a fissure extended through the body of the superior maxillary bone into the antrum, and upwards into the orbit; the malar bone was broken through, so that the zygomatic arch was flattened; the bones of the nose were driven in; and, in fact, it may be said that every bone upon that side of the face was fractured, and many comminuted.

"A short time after her admission, a person who had witnessed the accident brought the integuments which had been detached, so perfect, although thus separated, as still to preserve the perfect likeness; a drawing of which was immediately taken, and the parts preserved in our museum. I ordered the wound to be carefully washed and cleansed from all extraneous substances, and the portions of comminuted bone to be removed; and, as my great object was to keep down the tendency to inflammation, I directed that cold water should be kept constantly falling over the face, which was covered with lint, with a view of maintaining an equitable and low degree of temperature; at the same time, by constitutional means, subduing arterial action. The treatment had a most salutary effect, and, without a single bad symptom supervening, she got perfectly well; the cure, however, being necessarily protracted from the numerous portions of bone which exfoliated." (P. 60.)

Mr. Lonsdale, a very ingenious young surgeon, has invented an apparatus for the treatment of fracture of the lower jaw, an accident of which no mention is made in this work, excepting in the above quotation.

Passing over the rest of our author's comments on fractures, which are replete with useful matter and illustrative cases, but not sufficiently novel to interest our readers, we proceed to his account of the Diseases of Joints.

In a case of abscess of the knee-joint amputation was determined upon; but, as the patient was very weak, and suffering from diarrhœa, Mr. Cooper delayed the operation, and prescribed the following medicines:

"℞. Ammoniæ Carbonatis, gr. v.

Sodæ sub. carb., gr. xij.

Tinct. Cinch., ʒj.

Liq. Opii Sedat., gtt. vj.

Decoct. Cinchonæ, ℥iss. M.

fiat haust. bis in die sumend." (P. 150.)

We quote this prescription in order to censure the common but erroneous method of giving ammonia in vegetable infusions and decoctions: the combination forms a nauseous mess ill calculated to restore the tone of a jaded stomach; it is far better to give the Spir. Ammon. Arom. in plain water, to which the Tr. Cinnam. may be added *ad libitum.*

In speaking of disease of the hip-joint, Mr. Cooper says, "From the slowness of the progress, from the degree of constitutional irritation, from the length of time matter continues to flow after the abscess has been opened, and from the unhealthy kind of pus which is secreted, frequently offensive and discoloured, I am induced to believe that the disease of the hip, which terminates in abscess, most frequently begins in the bones of this articulation; and that the cartilages are not primarily affected, as has been stated by some writers." (P. 159.)

These reasons are not very satisfactory, especially as it is easy to suppose that, from the cartilages being primarily affected, the symptoms are at first slight, and that the violent symptoms detailed in the above quotation belong to the second stage of the disease. Mr. Brodie indeed distinctly says, "In whatever period of the disease the examination is made, the cartilages are found in a state of ulceration, but the morbid affections of the soft parts and bones vary very much; nor are they much altered from their natural state, except in the most advanced stage of the malady." (*Observ. on Dis. of the Joints,* 2d edit., p. 134.) Those who read German will thank us for informing them that an excellent and minute description of this disease is to be found in Jörg's "Kinderkrankheiten." Our author says,

"Our mode of treating the numerous children who apply to us for relief at Guy's Hospital, is first by local bleeding to subdue inflammation, by continued counter-irritation to divert the inflammatory action, and then attempt to improve the constitution by alterative and tonic medicines: thus, I generally order two grains of Hydrarg. c. cret., two of rhubarb, and five of carbonate of soda every night, and the following drops twice a day:

℞. Tinct. Cinchonæ, ℥i.
Tinct. Rhei. ℥ss.
Hydrarg. Oxym., gr. i. M.

Capt. gtts. xxv. bis in die, ex cyatho Infus. Anthem." (P. 160.)

Giving corrosive sublimate in Inf. Anthem. is worse than giving ammonia in Decoct. Cinchon., for the salt will be decomposed.

The following case is an interesting one:

"Mr. C., aged twenty-eight, consulted me in consequence of having observed, for a fortnight previous, that in walking he experienced a difficulty in raising the left foot clearly from the ground, and that he had an involuntary tendency to bend his body forwards during progression: these symptoms were unattended by pain, although he sometimes felt slight uneasiness on the inner side of the knee-joint, and a sense of weariness of the whole limb. On my asking him if there was any local cause to which he could attribute these symptoms, he mentioned that, seven weeks before, while in the performance of some athletic exercise, he felt a sudden darting pain, attended with a sensation of snapping, just below Poupart's ligament, in the centre of the inguinal region; and since that time he thinks he has constantly thrown more weight upon the other limb. On asking him to walk across my room, I observed that the diseased leg was in advance of the other, that his body was bent forwards to that side, and that in progression he used a stick, which he constantly employed in receiving the weight of his body, when the diseased limb should in its turn have borne it. On examining the pelvis, both in the anterior and posterior view, there was evident deformity: in front, the anterior and superior spinous process of the diseased side was seen depressed below that of the opposite; laterally, the trochanter major formed an obvious unnatural protuberance; and, behind, the gluteal region, from its projection, formed another diagnostic mark of the disease; the muscles of the thigh also were considerably wasted.

"Notwithstanding all these decided external signs of hip disease, still this gentleman was capable of following his usual avocations, although they called for the necessity of walking some distance daily. I explained to him the nature of his complaint, and with difficulty impressed upon his mind the necessity of his remaining for a length of time in a perfect state of rest; for I ascertained, by moving the joint, and striking the trochanter major so as to produce concussion of the head of the bone against the acetabulum, that there was not only inflammation of the synovial membrane, but that disease had commenced in the articular cartilage. His bowels were regular; pulse 100, and rather sharp; tongue white; and, like the pulse, indicating irritation. I requested him immediately he got home to be cupped to sixteen ounces on the hip, and afterwards to apply a full-sized blister over the joint, and to take the following medicines: ℞. Hydrarg. Submur., gr. iss.; Pulveris Opii, gr. ss. M. fiat pulv. omni nocte sumend. ℞. Iodinæ, gr. ss.; Potassæ Hydriod., ʒss.; Syr. Papav., ℥ss.; Inf. Gent. comp., ℥viij. M. capiat cochl. larg. ij. bis in die.

"The next day I found him in every respect worse; he complained of considerable pain in the hip-joint, extending down the inner side of the thigh to the knee, with inability to obtain ease from any position in which he could lie; I therefore increased his quantity of opium, ordered the blister to be kept open, and desired him to continue his mixture. In this state the patient remained, without any improvement, for nearly a month; during which period I affected his constitution by mercury, indicated by ptyalism, and gave him sarsaparilla, bark, and other tonic medicines, without however producing any beneficial effect. His pain was incessant; his constitution suffered materially, having some degree of hectic fever; and, as soon as he fell asleep, he experienced those sudden twitches of the muscles at the upper and inner part of the thigh,—the symptom, of all others, most indicative of the formation of matter: this result was further threatened by a tumour which was now evident in the inguinal region. These symptoms were, however, unattended by any distinct rigors. I ordered leeches to be applied to the tumour, and afterwards a belladonna plaster; and, as the bowels were at this period rather confined, attended with some disposition to nightly perspirations, prescribed the following medicine: ℞. Sulphatis Quininæ, gr. ij.; Magnes. Sulphat., ʒi.; Syrup. Aurantii, ʒi.; Liquoris Opii Sed., gtt. iv.; Infusi Rosæ comp., ʒx. M. fiat haus. bis in die sumend.

" I also passed a seton just below the trochanter major, including two inches of integument.

" From this period his symptoms were in some measure mitigated; his pain became less, his appetite improved, his nightly twitches diminished, and he was enabled to change the position of his limb with comparative ease to himself. From his mitigation of suffering he continued to improve in general health: he was able to sit upon the sofa, and, with crutches, even to walk about his room, so as to bear some slight weight upon the diseased limb, which gradually lost its apparent morbid length; the prominence of the gluteal region diminished; the muscles of the thigh regained their tone; and the patient may now be considered convalescent, after three months' illness.

" This case affords the interesting fact, either of all the symptoms of the formation of matter without the reality, or of the power of the restoration of the joint, even after matter has formed. The impression on my mind during the progress of the disease certainly was, that matter had formed: the circumscribed swelling, the nightly twitchings, the tendency to hectic fever, the intolerance of motion, and the general effect produced upon the constitution, when summed up, led to a most unfavourable prognosis; but continued rest, tonics, and generous diet restored his constitution, which in itself was sufficiently strong to undergo this severe ordeal. One peculiar symptom occurred in this case, that, when the patient first placed himself in the erect posture, he described a sensation in the hip-joint as if his capsular ligament was so filled with air as to prevent the head of the femur coming in contact with the acetabulum, communicating to him the sensation of the elasticity of an air-bag; nor did this morbid sensation cease for several weeks. This sensation must have occurred from some effusion: and, as I cannot believe it to be pus, from the sequel of the case, it may probably have been the effusion of synovia only which had produced it." (P. 161.)

Everyone knows that a dislocation of the humerus upon the dorsum of the scapula is one of the rarest accidents, and that the possibility of its occurrence has been denied by so great a surgeon as Boyer; we trust, therefore, that we shall not be considered as straying beyond the limits of a review in quoting at length the three cases of this anomaly which are given by our author.

" Case. Mr. G., a gentleman from Surrey, a very stout man, in the act of violently pushing a person from him, injured his right shoulder-joint, which led him to consult a surgeon, who did not discover the nature of the accident, but recommended him leeches, and all the means usually employed for the restoration of a contused joint. As however the use of the limb continued impaired, so much so that he could not raise his arm to his head, he came to town to consult Sir Astley Cooper, who not being at home, he applied to me. Upon examination, I found so much tumefaction, that I could not discover the nature of the injury, and desired him to call the next morning; when Sir Astley, raising the arm, for the purpose of examination, nearly perpendicular to the body, the head of the bone slipped forwards into the glenoid cavity, and thus the nature of the accident was made obvious only by the reduction of the dislocation.

"CASE. In a few days after it happened that a gentleman consulted Sir Astley Cooper, as a morning patient, who was the subject of a similar accident, and in whom the diagnostic marks were particularly clear. I at the moment was desired by Sir Astley to write down the principal features.

"The arm was directed inwards towards the side, giving it the appearance of fracture, and looking as if so carried for support. The roundness of the shoulder had lost its natural appearance, and the skin was gathered into folds in front of the acromion process, which was preternaturally prominent. On taking a posterior view of the shoulder, it was impossible to trace the spine of the scapula, in consequence of a fulness below and behind the acromion; and, upon raising the arm, the tumour in the fossa infraspinata moved obedient to the motions of the humerus, indicating the situation of the head of the bone; in tracing the long axis of which the eye was directed behind the glenoid cavity of the scapula. We then tried to reduce the dislocation by slight extension, drawing the arm outwards; but, failing in this attempt, Sir Astley Cooper raised the limb perpendicularly, and at the same time forcing it backwards behind the patient's head, the bone slipped into its place; being thus reduced precisely in a similar manner to the last-mentioned case. It was singular that two instances of so rare an accident should occur so closely together in the practice of one individual.

"These cases I was constantly in the habit of mentioning when lecturing on the subject of dislocation; and upon one occasion I received the following letter from an old pupil:

"CASE.

"My dear sir: Singularly enough, during your lecture of yesterday, while recapitulating the dislocations of the shoulder, and so fully describing the anatomy of the joint, I found, on my return home, that I had been sent for to an old woman, sixty years of age, living in Hen and Chicken court, Fleet street, who had fallen down in a fit. I immediately went to her, and found she had recovered from the fit, but complained dreadfully of pain in the shoulder, and of inability to move the arm. Upon a careful examination, which I was induced to make from its being so very different from what might have been expected from paralysis or neuralgic affection, I was soon led to infer that the articulatory surface of the humerus was thrown from the glenoid cavity of the scapula. Upon viewing the two shoulders, for the purpose of discovering the deviation from symmetry, it gave an appearance on the affected side as if the glenoid cavity was thrown forwards, and rendered particularly prominent. The whole arm appeared shorter and was directed forwards, but separated from the body; the head of the bone could be distinctly felt upon the dorsum of the scapula, producing a considerable tumour, and forming the grand diagnostic mark of the nature of the injury. I raised the arm perpendicularly to the body, in the manner I had just heard recommended by yourself; but, not being successful in returning the head of the bone into its place, and the attempt in this manner causing such considerable pain, I desisted, and proceeded in the following manner: The scapula being fixed, I made extension from the wrist in the direction of the displaced bone, (without placing my foot in the axilla,) for two or three minutes, while my assistant was directing the head of the bone forwards from the dorsum of the scapula, and in this way it readily slipped into its place.

"The coincidence of this accident with your lecture is somewhat singular.

"I am, my dear sir, your's, &c.

"Robert Dunn.

"2, *Fore Gate, St. Clement's Inn.*"

(P. 206.)

In speaking of Dislocations of the Patella, Mr. Cooper advances some extraordinary doctrines. He says,

"The dislocation *outwards* is the most frequent; it is, however, scarcely ever complete, being usually thrown upon the articular face of the external condyle, and not upon its outer surface. This accident is generally produced by a blow on the inner edge of the patella when the foot is everted, or in some persons who are very in-kneed the displacement may be produced merely by the action of the extensor muscles of the leg, in whom also there is generally a relaxation of the ligamentum patellæ, rendering them particularly liable to this accident. Boyer speaks of the form of the external condyle of the femur as a predisposing cause to the displacement of the patella, and mentions the cases of three military conscripts who were the subjects of this malformation, and had consequent displacement of the patella from their birth. If the dislocation be complete, the anterior surface of the patella must be turned outwards, its articulatory or posterior surface inwards, its internal edge must be directed forwards, while its outer edge has the opposite direction; and the knee presents, on viewing it anteriorly, a deep depression, pointing out how completely the displacement of the patella deprives the joint of its wonted protection." (P. 244.)

Now the fact is, that in a dislocation of the patella upon the outer condyle, the anterior surface of the bone is turned forwards and inwards, while the external edge sticks out, and is turned forwards and outwards; the inner edge rests upon the hollow behind the articular surface of the outer condyle.

This species of dislocation is produced by a blow upon the fore and inner part of the patella. The accident is not a frequent one. Its true nature, and the cause of the difficulty of reducing the dislocation in persons with large bones, as well as the way to get over this difficulty, were first explained by Mr. Mayo, in a letter printed in the "Medical Gazette" in 1827. When the bones are small, the dislocated patella is easily pushed back into its place; but when they are large, and deeply grooved, the edge of the patella is impacted in the groove of the outer condyle, and can be extricated from it only by bending the knee-joint to the utmost, when the ligamentum patellæ draws the bone out of the groove, and it snaps into its place. Mr. Bransby Cooper seems hardly aware of the difference between this kind of dislocation (the true dislocation from external violence,) and the one in which the bone lies with its articular surface flat upon the outer condyle. This latter species is common in children and young women, and the difficulty here is not in reducing the dislocation, but in preventing its eternal recurrence, or permanent continuance; so great is the facility with which the bone slips out and in.

In speaking of dislocation of the tibia backwards, Mr. Cooper remarks, that this accident "can hardly occur without being compound." (P. 247.) Rarely, we admit; yet an eminent surgeon of our acquaintance has seen the simple dislocation.

The last part of the "Surgical Essays" treats of wounds and injuries of the Abdomen, and is illustrated by some remarkable cases; as for example:

"About two years since, a man was brought into Guy's Hospital, in consequence of very severe injuries which he had received, while in the act of stealing lead from the top of a brewery, from which he fell. Upon examination, it was found that he had torn open an old scrotal hernia, and a considerable quantity of intestine protruded, and had remained exposed for nearly an hour; one of his thighs was also broken, and his left shoulder dislocated. The intestine was immediately returned into the cavity of the abdomen, and the edges of the wound brought together by the uninterrupted suture; the fractured thigh was placed in splints, and the dislocated shoulder reduced, which was accomplished with much more than usual facility, in consequence of the state of collapse of the patient from his abdominal injury. His pulse being feeble, the surface of his body cold, and his respiration difficult, julep ammon. was administered, and bottles of hot water applied to his feet, for the purpose of producing re-action, which was no sooner effected than pain in the abdomen came on, for which leeches were applied, and calomel with opium given, for the purpose of allaying his pain: all the symptoms, however, rapidly increased in urgency, and in fifteen hours after his admission he died.

"Upon examination of his body, it was found that he had been the subject of severe peritonitis, demonstrable from the quantity of coagulable lymph which was poured out; the portion of intestine which had protruded had not been ruptured, nor were there any signs by which it could be known from the rest of the intestines, but from a slight degree of thickening, probably from its frequent descent into its old hernial sac. The diaphragm was found ruptured, and a considerable portion of the stomach protruded into the chest; a circumstance of which there was no suspicion from the symptoms during life." (P. 263.)

Our author has a few observations on the treatment of Strangulated Hernia, and agrees with Mr. Key in thinking that we do well, when we can, to return the contents of the hernial sac into the abdomen, without opening it. He says,

"As Mr. Key very properly asks, who would hesitate to return a strangulated hernia by the employment of the taxis, instead of cutting down upon the part, and first examining the intestine? unless indeed there were, as there might be, outward signs of sphacelus or rupture from some other cause. I must also say, that I believe it will frequently be found, from adhesions and other causes, that the contents of a hernial sac cannot be returned, unless the peritoneum be opened; but, however true this may be, and to whatever extent, still it does not diminish the value of the proposed step, where the performance of it is admissible.

"In the still further aggravation of injuries to the abdomen, it may happen not only that the parietes should be wounded, and its contents protrude, but the viscera themselves may also be injured; and there can scarcely be in surgery a greater difficulty than in this kind of accident; a difficulty which frequently presents itself in cases of strangulated hernia, when the intestine has given way either from sloughing or ulceration. What is the treatment to be pursued in these cases? Either is the intestine to be returned into the cavity of the abdomen, and the edges of its wound left as near as possible to the opening through the parietes, leaving it to nature alone to secure it there by the adhesive inflammation? or is the surgeon, by means of suture through the bowel itself, or the mesentery, to fix it there? From my own experience, founded upon dissection, as well as upon experiments on the lower animals, I am inclined to come to the following conclusion, and to recommend that the means to be employed should be regulated by the nature of the injury inflicted upon the intestine. If the wound of the intestine be through the whole of its calibre, extending to the mesentery, or, at any rate, implicating a large portion of the cylinder, I then should recommend suture: dangerous as it may seem to be, it is yet to be considered the only means left to secure the position of the intestine near to the outer wound; but if, on the contrary, when the wound extends only through a small portion of the calibre, the suture need not be used; for nature immediately, by the adhesive inflammation, not only unites the edges of the wound of the intestine and parietes together, but also by the same process provides a perfect barrier to the discharge of feculent matter into the cavity of the abdomen. It not unfrequently happens, where the opening into the bowel is small, that its calibre is immediately re-established by the union of its edges to the parietes of the abdomen, without communicating with the external wound; generally, however, the mode of reparation is more protracted, and an artificial anus is first produced, the obliteration of which becomes the subject of further treatment, and usually, by the judicious application of mechanical and constitutional means, it may be effected.

"I have made several experiments in the endeavour to draw some just inference as to the best mode of treatment in cases of protruding wounds of the intestine; but I fear, from the experiments being made upon lower animals, there is much danger in drawing too hasty deductions from any supposed analogy between the condition in which the human subject would be placed under the same circumstances with them; at any rate, I discovered that the difficulty in preventing the escape of fæces into the abdomen was exactly in proportion to the size of the opening in the intestine; and would recommend that where the opening was large, whether from accident or disease, as in cases of strangulated hernia, that sutures should be employed in such a manner as to prevent any portion of the wound opening into the cavity of the abdomen. I have never seen an accident of wounded parietes of the abdomen, with protrusion of injured intestine, except in cases of gun-shot wounds, which were removed from my care immediately, and have left no further impression upon my mind, than the recollection of the immediate prostration which follows such accidents. I shall, however, describe two or three cases of strangulated hernia, in which the intestine had been found either sphacelated or ulcerated; and leading, therefore, to the same surgical considerations as belong to this class of abdominal injury.

"Case. James Crew, aged fifty-six, was admitted into Guy's Hospital, the subject of a strangulated scrotal hernia, from which he had suffered with insuperable constipation for nine days. The patient was immediately put in a warm bath, had a small quantity of blood taken from his arm, and the taxis employed without any benefit: the operation was therefore immediately proposed and consented to. Upon laying open the sac, a large portion of crispy, dark-coloured omentum was exposed, covering a considerable knuckle of intestine, which adhered to the sac by recent depositions of lymph, but was readily separated from it. I then freely divided the stricture, and on examining the state of the strangulated bowel found it dark-coloured, but its physical qualities did not in my mind indicate sufficient loss of vital power as to induce me to leave it within the hernial sac; feeling then, as I now do, convinced, that if there be any chance of the restoration of the bowel, that chance is much increased by its return into its natural cavity, which I therefore effected, removing at the same time the protruded omentum, which, from its consolidation, would have probably acted as an extraneous body. The intestine was left as near as possible to the mouth of the sac, and the wound being dressed, the patient was put to bed, when he immediately began to describe a sensation of cold and shivering, his pulse was feeble, and his body bedewed with a cold clammy perspiration; bottles of hot water were applied to his feet, and brandy, with julep ammoniæ, given. From this treatment he rallied, and seemed to be easier; but in the middle of the night he was seized with a violent pain in the abdomen; collapse came on about five in the morning, when he died.

"Upon examination of the body after death, the pelvis was found nearly filled with fæculent matter; and upon searching for the opening in the intestine, through which it had escaped, a small ulcerated orifice was found, accounting at once for the sudden dissolution of the patient. The character of an opening in an intestine will always shew whether it has been the result of violence or of disease; if of violence, the opening presents a thickened protruded edge, produced by the eversion of the mucous membrane; while, on the contrary, if it be ulcerated, the opening presents a thin edge, terminating by peritoneum instead of mucous membrane, in consequence of the much greater rapidity with which ulceration goes on in the mucous, than the serous membranes.

"I have described this case, on account of its being instructive in pointing out the necessity of learning the external marks by which an intestine shews its degree of disorganization; words can hardly express what experience teaches, but the peculiar elasticity, thickness, natural colour, and degree of warmth, are the means by which this important point is to be ascertained. One of the most certain signs, perhaps, in cases of strangulated hernia, as to the degree of vitality of intestine, is its power of regaining its colour as soon as the stricture is divided, a circumstance which points out at once the propriety of returning such intestine into the abdomen." (P. 265.)

We have already given our opinion of Mr. Cooper's book, considered as a surgical work: it is sober and sensible, contains many interesting cases, and, in spite of some errors, will be very useful to beginners. But there is another point of view from which these essays may be contemplated, and which would compel us to use the language of unmixed censure. Their style is slovenly and ungrammatical to the last degree, and we should ill discharge the office of unbiassed censors if we suffered any consideration to shield their author from reproach. We promised, indeed, that in doubtful cases we should incline to mercy, rather than rigid equity; but here the case is too clear, and, when critical justice demands her victim, it shall never be said that we performed our duty with the reluctance of partiality. Sentences like the following ones abound in this book:

"We also find that persons who die a sudden death, and where life has been destroyed by a narcotic poison, that the pupils are contracted." (P. 19.)

"With so extensive an injury, no hopes could be entertained of recovery; but yet it may appear to some, after the principles which have been laid down for the treatment of bleeding from the lungs, and for emphysema, whether or not the lancet might have been used in this case," &c. (P. 53.)

"Of all the structures entering into the composition of joints, synovial membrane is the most frequently affected; and this tendency to inflammation has been attributed to the similarity, if not to the identity, between them and the serous membranes." (P. 140.)

"When ulceration has taken place, and the articular cartilage is removed by the process of absorption, the bone of the joint becomes diseased, which advances more rapidly than the cartilage," &c. (P. 145.)

"An accident to which the inferior extremities of the bones of the fore-arm are liable has lately been noticed by Sir Astley Cooper, and written on by a French surgeon of considerable eminence, in one of the Paris journals, is a fracture through the lower extremity of the radius, in the situation of the epiphysis in the adult, and through the epiphysis at earlier periods of life." (P. 219.)

"My colleague, Mr. Key, admitted a patient a few days since," &c. (P. 247.) A few days before what period? Surely this is a bit of raw, undigested note-book, and may be called a specimen of the *lienteric* style.

We had marked many more passages for public exposure, but we are tired of this ungracious task. After all, these blemishes do not detract from Mr. Cooper's surgical skill; and, though we certainly should not ask his advice had we broken Priscian's head, we should be too happy in his charitable aid had we broken our own.

